# Total Flavonoids Content in the Raw Material and Aqueous Extractives from *Bauhinia monandra* Kurz (Caesalpiniaceae)

**DOI:** 10.1100/2012/923462

**Published:** 2012-06-04

**Authors:** Ana Josane Dantas Fernandes, Magda Rhayanny Assunção Ferreira, Karina Perrelli Randau, Tatiane Pereira de Souza, Luiz Alberto Lira Soares

**Affiliations:** ^1^Programa de Pós-Graduação em Ciências Farmacêuticas, Departamento de Farmácia, UFRN, Avenida Gal. Cordeiro de Farias s/n, 59010-180 Natal, RN, Brazil; ^2^Programa de Pós-Graduação em Ciências Farmacêuticas, Departamento de Ciências Farmacêuticas, UFPE, Rua Prof. Artur de Sá s/n, 50740-521 Recife, PE, Brazil; ^3^Departamento de Medicamentos e Alimentos, Universidade Federal do Amazonas, Rua Alexandre Amorim 330, Aparecida, 69010-300 Manaus, AM, Brazil

## Abstract

The aim of this work was to evaluate the spectrophotometric methodology for determining the total flavonoid content (TFC) in herbal drug and derived products from *Bauhinia monandra* Kurz. Several analytical parameters from this method grounded on the complex formed between flavonoids and AlCl_3_ were evaluated such as herbal amount (0.25 to 1.25 g); solvent composition (ethanol 40 to 80%, v/v); as well as the reaction time and AlCl_3_ concentration (2 to 9%, w/v). The method was adjusted to aqueous extractives and its performance studied through precision, linearity and preliminary robustness. The results showed an important dependence of the method response from reaction time, AlCl_3_ concentration, sample amount, and solvent mixture. After choosing the optimized condition, the method was applied for the matrixes (herbal material and extractives), showing precision lower than 5% (for both parameters repeatability and intermediate precision), coefficient of determination higher than 0.99, and no important influence could be observed for slight variations from wavelength or AlCl_3_ concentration. Thus, it could be concluded that the evaluated analytical procedure was suitable to quantify the total flavonoid content in raw material and aqueous extractives from leaves of *B. monandra*.

## 1. Introduction

*Bauhinia *species, such as *B. monandra* Kurz, are popularly known in Brazil as “Pata-de-Vaca” and are widely used in traditional medicine as antidiabetic or antioxidant agent. Although studies showed such activities, they were not still elucidated which group(s) of compound(s) is/are responsible for such properties [1, 2]. In the literature the presence of flavonoids in the leaves of vegetable is related [1]. Additionally, the several pharmacologic properties and wide occurrence in the vegetable kingdom with restricted distribution inside of an order, family, or gender; these compounds have been widely used as chemical markers of first choice for several active herbal drugs [3–9].

The general technique most employed for quantification of flavonoids is based on the spectrophotometric determination of complex flavonoid-AlCl_3_, which provides a bathochromic displacement and the hyperchromic effect [10]. Although present in official codes, the method proposed initially by Christ and Müller [11] presents several analytic limitations that can be configured as significant source of error. Initially, the technique was developed for analysis of herbal materials containing O-glycoside flavonoids, the ones which, after acid hydrolise and extraction with organic solvent, are quantified starting from the compound formed between the flavonoid aglycones and AlCl_3_. However, the technique has been indiscriminately used for vegetable species containing C-glycosides flavonoids. How they do not undergo acid hydrolyze, they are discarded in the aqueous phase during the liquid-liquid extraction due to the polarity from glucosyl residue [5, 6, 12].

Additionally, several factors have been revealed as crucial during the formation of the flavonoid-AlCl_3_ complexes such as: the reaction time, the concentration of the reagent (AlCl_3_ and flavonoid content/plant material), and the chemical structure of the polyphenol. Likewise, the study of these parameters becomes fundamental during the development of the method [6, 12]. Thus, the use of multivariate approach is quite suitable for satisfactory evaluation of this kind of challenge. Experimental designs are widely used multivariate tools for the systematic and effective evaluation of simultaneously modifications of several variables in a specific system [13, 14]. Special attention has been direct to central composite design (CCD). This second-order design is the most often employed to study and optimize several systems such as: reactional conditions [12]; operational parameters [15]; formulation compositions [16]; method stability [17], and/or extractive performance [18, 19]. With this kind of second-order design, it is possible to create response surfaces that allow the ranking of each variable according to its significance on the studied responses [13, 14, 20]. Therefore, with reduced time and experimental effort, it may be possible to predict what experimental condition will produce a desired or optimum response.

Therefore, the objective of this work was the evaluation of a methodology for quantification of total flavonoids without acid hydrolyzes, in the herbal material and aqueous extract from leaves of *B. monandra*.

## 2. Material and Methods

### 2.1. Materials

The leaves of the *Bauhinia monandra* Kurz were collected in November of 2004, in Campina Grande, PB, and taxonomically identified. Voucher specimen was identified by Dra. Maria Iracema Bezerra Loyola and deposited at Department of Biology/UFRN under registration number 2611. The dried and ground material was used in all experiments. All reagents used were grade proanalysis.

### 2.2. Methods

#### 2.2.1. Wavelength Selection

**Herbal Material**

For the determination of the maximum of absorption, a sample of 0.5 g of the herbal material (*d*_50_ = 0.615 mm) was submitted to a general procedure for flavonoids quantification using ethanol 80% (v/v) as liquid extractor. The UV-Vis spectra (200 to 500 nm) was obtained after 30 minutes of complex with AlCl_3_ 5% (w/v) in hydroalcoholic solution 80% (v/v).

**Extractives**

The spectrum from 200 to 500 nm was obtained in spectrophotometer (Cary 1E, Varian, Japan) 30 minutes after addition of 2.0 mL of AlCl_3_ 2.5% (w/v) to the diluted aqueous extractives (dilution = 1/208.33). The same sample without AlCl_3_ was used as compensation solution.

#### 2.2.2. Optimization of Reaction Parameters for Formation of Flavonoid-AlCl_3_ Complex

The influences of reaction time and concentration of AlCl_3_ were studied through an experimental design 2^2^ (AlCl_3_ = 2.5 or 7.5%, w/v; time = 15 or 35 min), added of 3 central points (AlCl_3_ = 5%, w/v; time = 25 min) and 4 star points (AlCl_3_ = 1.46 or 8.54%, w/v; time = 10.86 or 39.14 min) [12]. Each point represents an average of three determinations. The results were adjusted by PLS to a mathematical model, which was evaluated statistically and used to generate response surface.

The independent variable factors for experimental design were the concentration of AlCl_3_ and time. The dependent variable (response) was the absorbance. To compare the effect of factors, their values were coded (Table 1). A second-order model was established for the responses by PLS (1). The validation of the mathematical model was performed through analysis of variance, multiple-correlation coefficients, and estimation of the lack of fit using STATISTICA 6.0 (Statsoft, USA):

(1) $$\begin{array}{cc} Y & =b_{0}+b_{1}\cdot X_{1}+b_{2}\cdot X_{2}+b_{12}\cdot X_{1}\cdot X_{2}\\ & \;+b_{11}\cdot {\left(X_{1}\right)}^{2}+b_{22}\cdot {\left(X_{2}\right)}^{2}. \end{array}$$

#### 2.2.3. General Procedure for Total Flavonoids Quantification in Herbal Material

The sample of raw material was transferred to a round-bottom flask of 250 mL and added of 30.0 mL from hydroalcoholic solution. The mixture was heated up in water bath, under reflux, by 30 min. After cooling at room temperature, the extractive was filtered to a volumetric flask of 100 mL through small amount of cotton. The residue of herbal drug and the cotton were resubmitted to reflux with more 30.0 mL from the same solvent by 30 min. The procedure was repeated once more. The volume was completed to 100 mL originating the stock solution (SS). 1.0 mL from SS was transferred to a 25 mL volumetric flask of, added of 2.0 mL from AlCl_3_ solution and the volume was completed with distilled water (probe solution, PS). The same procedure was repeated without the addition of AlCl_3_ for preparation of contrast solution (CS). The absorbance of PS against CS was determined in spectrophotometer at 410 nm.

The result express as the percentage of total flavonoid content (TFC), calculated as rutin, through (2), and it represents the average of three determinations

(2) $$\text{TFC}=\frac{A\cdot \text{DF}}{A_{1\text{cm}}^{1\%}\cdot \left(w-\mathrm{ld}\right)},$$

where *A* = absorbance (A.U.); DF = dilution factor; *w* = mass of plant material (g); ld⁡ = loss on drying of plant material (8%, w/w);  *A*_1cm_^1%^ = specific absorption for rutin-AlCl_3_ complex (259,4).

**Evaluation of the Solvent Concentration**

0.5 g of raw material was extract with ethanol 40, 60, 80, or 100% (v/v), according to the general procedure for herbal material. The absorbance was determined by spectrophotometry at 410 nm after 25 min using AlCl_3_ 5% (w/v). The total flavonoid content (TFC) was expressed by the average of three determinations.

**Evaluation of Herbal Material Concentration**

Samples of the raw material ranged from 0.25 to 1.25 g were submitted to the general procedure of quantification using ethanol 80% (v/v) as solvent. The absorbance was determined by spectrophotometry at 410 nm after 25 min using AlCl_3_ 5% (w/v). The results express the average of three determinations.

#### 2.2.4. General Procedure for Total Flavonoids Quantification in the Aqueous Extractives

The sample was constituted by aqueous extractive prepared by decoction for 10 min at plant : solvent proportion of 15 : 100 (w/v). The solution was filtered and the volume completed to 100 mL with distilled water (extractive solution, ES). Aliquot of 3.0 mL from ES was diluted to 100 mL with water distilled in volumetric flask (stock solution, SS). Another aliquot of 4.0 mL of SS was transferred to a 25 mL volumetric flask and added of 2 solution mL from AlCl_3_ 2.5% (w/v) (probe solution, PS). The same procedure was repeated without the addition of AlCl_3_ for preparation of the contrast solution (CS). The absorbance of PS against CS was determined in spectrophotometer in 410 nm after 15 minutes of reaction. The total flavonoid content was calculated according to (2).

#### 2.2.5. Calibration Curve for Aqueous Extractives

The aqueous extractive from leaves of *B. monandra* was filtered through filter paper and diluted with distillated water to concentrations ranged from 0.3 to 1.44 mg/mL. 2.0 mL of AlCl_3_ 2.5% (w/v) were added to each diluted solution and the solution was made up to 25.0 mL with distillated water. The absorbance was measured at 410 nm after 15 min, using the diluted solution without AlCl_3_ as contrast solution. The calibration curve was made by linear regression and the results represented the average of three determinations to each concentration.

#### 2.2.6. Repeatability and Intermediary Precision

The repeatability was evaluated in triplicate on the same day for three samples, while the intermediary precision was assessed for two consecutive days. The dates were expressed for relative standard deviation (RSD%) [21].

#### 2.2.7. Preliminary Robustness

The robustness of the method was evaluated through the determination of the absorbance of the solution extractive complexed with aluminum chloride in the concentrations of 5.0 ± 0.5% (w/v) and absorbance determinate at 410 ± 2 nm. Each determination represents average of three determinations [21]. 

## 3. Results and Discussion

### 3.1. Wavelength Selection for Herbal Drug and Extractives

**Figure 1:**
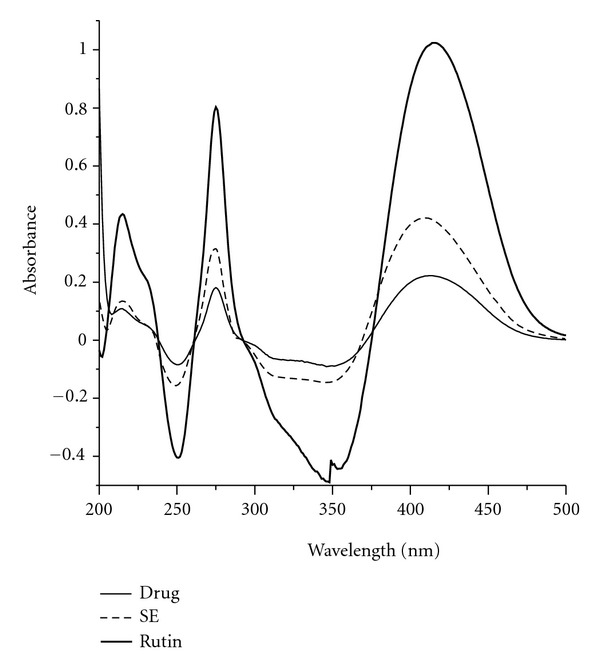
Spectrum for AlCl_3_-flavonoid complexes: herbal material (drug), aqueous extractive (SE) and standard (rutin).

The UV-spectrum for herbal drug, aqueous extractive, and rutin after reaction with AlCl_3_ are presented in Figure 1. A very similar UV-profile was observed for all sample tested and three maximums were observed. Due to the higher specificity of absorptions at wavelength corresponding to the band I derived from cynamoil moiety of flavonoids, the determinations were performed at 410 nm for both herbal material and respective aqueous extractives.

### 3.2. Total Flavonoid Content in Herbal Material

#### 3.2.1. Reactional Conditions for Formation of Flavonoid-AlCl_3_ Complex in Herbal Material

The results for method performance upon several reaction times and/or AlCl_3_ concentrations are shown in Table 1. The experimental data for herbal material were used to generate second-order models for each dependent variable. Due to the lack-of-fit test was not significant, the experimental variations could be attributed only to a randomized error. Thus, the fitted models provided an adequate approximation of the true values, and no violations of the model assumptions occurred.

**Figure 2:**
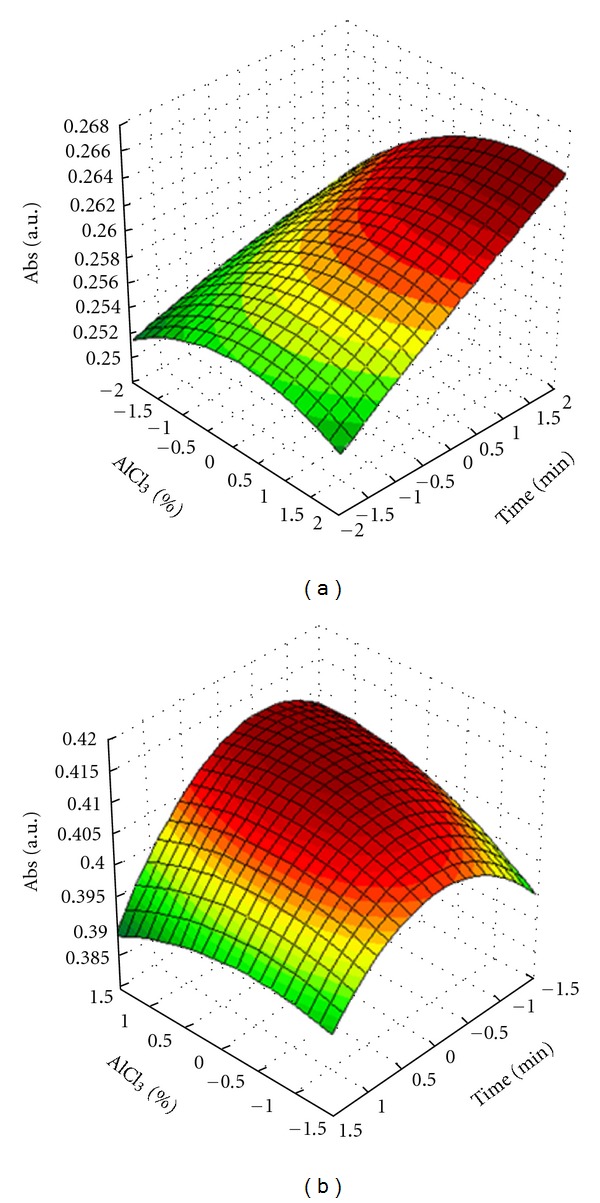
Effects of reaction time and AlCl_3_ concentration on the spectrophotometric responses for the analytical methodology applied to drug material (a) and aqueous extractives (b) from leaves of *B. monandra*.

The mathematical models that explain each surface are calculated by (1) and the data used to generate response surface (Figure 2(a)).

The ANOVA was significant for both factors, concentration of AlCl_3_ and time, demonstrating that the variation of these significantly influence the response studied (Table 2).

In agreement with the Table 3, the *t*-test for the coefficients revealed statistical importance for lineal terms of variables concentration of AlCl_3_ and, mainly, time. The quadratic terms exercised negative effect on the response only being significant the factor AlCl_3_.

In that way, the elected conditions for determining a higher response for total flavonoid content in plant material were 5% (m/v) of AlCl_3_ and 25 minutes of reaction.

#### 3.2.2. Influence of Solvent and Plant Concentration on the Assay Performance for the Herbal Material

**Figure 3:**
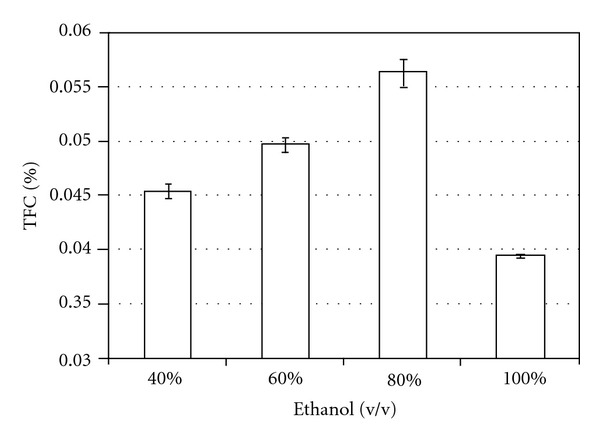
Influence of extractive solvent on the total flavonoid content (TFC) from drug material.

The total flavonoids content, calculated for drug material when 0.5 g was extracted with ethanolic solutions in different concentrations (40 to 100%), showed that alcohol 80% (v/v) determines a higher response for the method (Figure 3).

**Figure 4:**
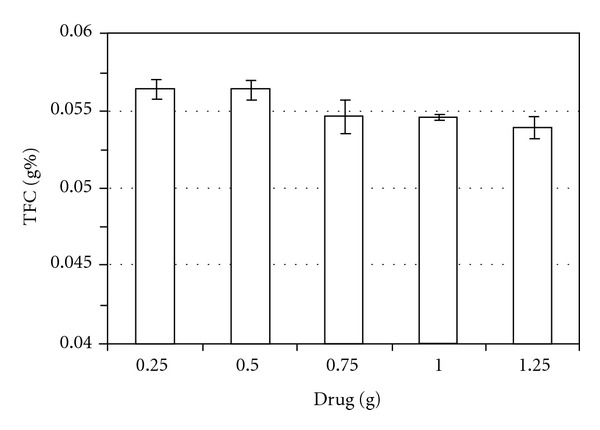
Influence of drug amount on the total flavonoid content (TFC) from drug material.

Selected ethanol 80% as better concentration of solvent extractor, the influence of proportion plant : solvent was evaluated using samples ranged from 0.25 to 1.25 g. Analyzing the results, it was verified that the best parameters, were 0.5 g of herbal material and 80% (v/v) of ethanol, where the higher response was obtained. The apparent decrease of flavonoid content with increase of plant proportion can be explained by the possible saturation of solvent or method failure (Figure 4) [22].

#### 3.2.3. Repeatability and Intermediary Precision

The data for procedure precision by repeatability and intermediary precision showed that the method was precise due to the lower relative standard deviation (RSD%) for both parameters. The repeatability showed relative standard deviation of 2.5%, while no statistical difference could be observed for intermediary precision, although the method was appraised on different days. Thus the method could be considered in according with the legal requirements for analytical performance of procedures for evaluation of phytopharmaceuticals and phytomedicines (Table 4).

### 3.3. Total Flavonoid Content in the Aqueous Extractives

#### 3.3.1. Reactional Conditions for Formation of Flavonoid-AlCl_3_ Complex in Aqueous Extractives

The statistical analysis by ANOVA (Table 2) and *t*-test for coefficients of equation (Table 3), showed no significant variation for different terms of equation. Although the literature relates that the reaction time and concentration of AlCl_3_ are critical for flavonoids determination and should be optimized for each matrix vegetable, it was not the discovery for the aqueous extractive solution of *B. monandra*. Anyway, the surface generated by experimental data suggested a tendency to an optimal conditions at 2.5% (w/v) of AlCl3 after 15 minutes for reaction time, which allow to determine the flavonoids content without risk of method failure (Figure 2(b)).

#### 3.3.2. Method Evaluation for Aqueous Extractives

**Figure 5:**
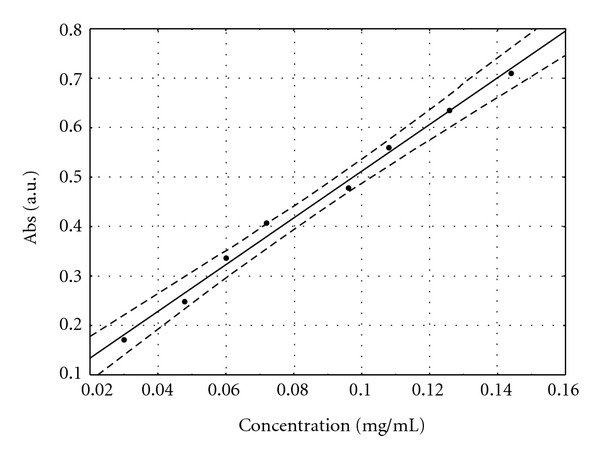
Calibration curve for aqueous extractives for flavonoids-AlCl_3_ complex from leaves of *B. monandra*.

The calibration curve was obtained by the absorbance concentrations (mg/mL) using eight dilutions. The regression analysis was performed and the resulting equation was “Abs = 0.0396 + 4.73*x*.” The coefficient of determination for standard curves were greater than 0.99 (*R* ^2^ = 0.998). Thus, the calculated straight line could explain more than 99% of the experimental data (Figure 5).

Table 4 shows the precision of method. This presented RSD of 2.68% for repeatability, with RSD intrasample varying from 1.53 to 3.19%. This improvement of the variation between days can be explained by the introduction of different extracts for each day of analysis [19].

Regarding the robustness of the method, the analysis by two-way ANOVA with repetition revealed that the response did not undergo any significant interference when small changes were inserted in the concentrations of AlCl_3_ and/or in the maximum wavelength (Table 5). This became, therefore, a robust method for determination of TFC in aqueous extractives of *B. monandra*.

## 4. Conclusions

This study showed that the method initially developed by Schmidt and Ortega [6] for aerial parts of Passiflora was also appropriate for the determination of the total flavonoids content (TFC) in the herbal material and aqueous extractive from *B. monandra* Kurz. However, before adoption of the proposed procedure, several tests were performed in order to obtain the optimized analytical conditions. After that, the results allowed to conclude that the proposed procedure is simple, fast and precise, and can be used to support the quality evaluation of raw material and f the aqueous extractives from *B. monandra*.

## Figures and Tables

**Table 1:** Matrix of central composite design (CCD) applied to evaluate the influence of reaction time and AlCl_3_ concentration on the method responses (absorbance).

	Coded variables		Natural variables		Method response	
Experiment	Time (min)	AlCl_3_ (%)	Time (min)	AlCl_3_ (%)	Drug (A.U.)	Extractives (A.U.)
1	−1	−1	15	2.5	0.255	0.400
2	1	−1	35	2.5	0.258	0.398
3	−1	1	15	7.5	0.258	0.409
4	1	1	35	7.5	0.264	0.400
5	0	0	25	5	0.258	0.409
6	0	0	25	5	0.258	0.410
7	0	0	25	5	0.256	0.409
8	0	−1.414	25	1.46	0.262	0.397
9	0	1.414	25	8.54	0.259	0.413
10	−1.414	0	10.86	5	0.260	0.405
11	1.414	0	39.14	5	0.260	0.414

A.U.: absorbance units.

**Table 2:** ANOVA for CCD applied to evaluate the influence of reaction time and AlCl_3_ concentration on the method responses for both drug material and extractives from leaves of *B. monandra. *

Source	*F*_(Drug)_	*F*_(Extractives)_
AlCl_3_ (linear)	546.76*	0.68
AlCl_3_ (quadratic)	131.85*	0.63
Time (linear)	1654.53*	3.59
Time (quadratic)	2.351	5.69
Interaction (time × AlCl_3_)	38.36*	0.64

*Significant for *α* = 0.05.

**Table 3:** *t*-test for effects of factors on the method response for drug and extractive solution from leaves of *B. monandra*.

Source	*t*_(Drug)_	*t*_(Extractives)_
Average	3092.65*	143.01
AlCl_3_ (linear)	23.38*	0.83
AlCl_3_ (quadratic)	−11.48*	−0.80
Time (linear)	40.68*	−1.90
Time (quadratic)	−1.53	−2.40
Interaction (time *×* AlCl_3_)	6.19	−0.80

*Significant for *α* = 0.05.

**Table 4:** Repeatability and intermediate precision of the method for drug material and aqueous extractive from leaves of *B. monandra *(results in absorbance).

	Drug			Aqueous extractives		
	Day 1	Day 2	Repet.	Day 1	Day 2	Repet.
Average	0.266	0.276	0.271	0.412	0.417	0.414
SD	0.004	0.004	0.006	0.006	0.13	0.011
RSD%	1.701	1.739	2.50	1.53	3.19	2.68

**Table 5:** ANOVA for the preliminary robustness of the procedure for TFC in aqueous extracts from leaves of *B. monandra*.

Source of variation	*F*	*F*_critical_
AlCl_3_ (%)	0.29	3.55
*λ* (nm)	1.56	
Interation	1.23	

*Significant for *α* = 0.05.
